# Glucagon-like Peptide-1 Receptor Activation Reduces Pulmonary Vein Arrhythmogenesis and Regulates Calcium Homeostasis

**DOI:** 10.3390/ijms241713100

**Published:** 2023-08-23

**Authors:** Chao-Shun Chan, Fong-Jhih Lin, Yao-Chang Chen, Yung-Kuo Lin, Satoshi Higa, Shih-Ann Chen, Yi-Jen Chen

**Affiliations:** 1 Division of Cardiology, Department of Internal Medicine, School of Medicine, College of Medicine, Taipei Medical University, Taipei 11031, Taiwan; chan_chao_shun@yahoo.com.tw (C.-S.C.); yklin213@yahoo.com.tw (Y.-K.L.); 2 Division of Cardiology, Department of Internal Medicine, Taipei Medical University Hospital, Taipei Medical University, Taipei 11031, Taiwan; 3 Graduate Institute of Medical Sciences, College of Medicine, Taipei Medical University, Taipei 11031, Taiwan; hotelfather@yahoo.com.tw; 4 Department of Biomedical Engineering, National Defense Medical Center, Taipei 11490, Taiwan; bme02@ndmctsgh.edu.tw; 5 Division of Cardiology, Department of Internal Medicine, Wan-Fang Hospital, Taipei Medical University, Taipei 11696, Taiwan; 6 Cardiac Electrophysiology and Pacing Laboratory, Division of Cardiovascular Medicine, Makiminato Central Hospital, Okinawa 9012131, Japan; sa_higa@yahoo.co.jp; 7 Heart Rhythm Center, Division of Cardiology, Department of Medicine, Taipei Veterans General Hospital, Taipei 11217, Taiwan; epsachen@ms41.hinet.net; 8 Institute of Clinical Medicine and Faculty of Medicine, National Yang Ming Chiao Tung University, Taipei 11217, Taiwan; 9 Cardiovascular Center, Taichung Veterans General Hospital, Taichung 40705, Taiwan; 10 Graduate Institute of Clinical Medicine, College of Medicine, Taipei Medical University, Taipei 11031, Taiwan; 11 Cardiovascular Research Center, Wan-Fang Hospital, Taipei Medical University, Taipei 11696, Taiwan

**Keywords:** glucagon-like peptide-1 receptor agonist, atrial fibrillation, pulmonary vein

## Abstract

Glucagon-like peptide-1 (GLP-1) receptor agonists are associated with reduced atrial fibrillation risk, but the mechanisms underlying this association remain unclear. The GLP-1 receptor agonist directly impacts cardiac Ca^2+^ homeostasis, which is crucial in pulmonary vein (PV, the initiator of atrial fibrillation) arrhythmogenesis. This study investigated the effects of the GLP-1 receptor agonist on PV electrophysiology and Ca^2+^ homeostasis and elucidated the potential underlying mechanisms. Conventional microelectrodes and whole-cell patch clamp techniques were employed in rabbit PV tissues and single PV cardiomyocytes before and after GLP-1 (7-36) amide, a GLP-1 receptor agonist. Evaluations were conducted both with and without pretreatment with H89 (10 μM, an inhibitor of protein kinase A, PKA), KN93 (1 μM, an inhibitor of Ca^2+^/calmodulin-dependent protein kinase II, CaMKII), and KB-R7943 (10 μM, an inhibitor of Na^+^/Ca^2+^ exchanger, NCX). Results showed that GLP-1 (7-36) amide (at concentrations of 1, 10, and 100 nM) reduced PV spontaneous activity in a concentration-dependent manner without affecting sinoatrial node electrical activity. In single-cell experiments, GLP-1 (7-36) amide (at 10 nM) reduced L-type Ca^2+^ current, NCX current, and late Na^+^ current in PV cardiomyocytes without altering Na^+^ current. Additionally, GLP-1 (7-36) amide (at 10 nM) increased sarcoplasmic reticulum Ca^2+^ content in PV cardiomyocytes. Furthermore, the antiarrhythmic effects of GLP-1 (7-36) amide on PV automaticity were diminished when pretreated with H89, KN93, or KB-R7943. This suggests that the GLP-1 receptor agonist may exert its antiarrhythmic potential by regulating PKA, CaMKII, and NCX activity, as well as modulating intracellular Ca^2+^ homeostasis, thereby reducing PV arrhythmogenesis.

## 1. Introduction

Diabetes and atrial fibrillation (AF) are becoming more prevalent globally and often occur together due to shared risk factors like obesity and hypertension [1,2]. In patients with diabetes, the prevalence of AF is twice as high compared to those without diabetes [3], consequently increasing the risk of stroke in AF patients [4]. Several factors have been suggested to contribute to an increased risk of AF in diabetic patients, including inflammation, oxidative stress, advanced glycation end products, impaired cardiac energy metabolism, electrical and structural remodeling, and autonomic dysfunction in the atria [5,6,7]. It is believed that these processes collectively heighten the likelihood of AF development in individuals with diabetes. The impact of antidiabetic drugs on the risk of AF is crucial, and it has been observed that intensive glycemic control does not effectively reduce new-onset AF [4]. Furthermore, different antidiabetic agents may have varying effects on the pathogenesis of AF [8]. Among the antidiabetic drugs, glucagon-like peptide-1 (GLP-1) receptor agonists have been shown to have dual benefits, exhibiting glucose-lowering and cardioprotective effects while also reducing the likelihood of non-fatal myocardial infarction, stroke, and cardiovascular death in diabetic patients [9,10,11,12]. However, it is worth noting that a meta-analysis reported a consistent increase in heart rate (from two to eight beats per minute) in patients receiving GLP-1 receptor agonist treatment [13]. This increase in heart rate has been attributed to the modulation of the autonomic nervous system [14,15,16] and may contribute to the pathogenesis of AF. On the other hand, several meta-analyses have indicated that GLP-1 receptor agonists are associated with a lower risk of AF compared to other glucose-lowering agents [17,18]. This suggests a potential protective effect of GLP-1 receptor agonists against AF development.

AF is linked to disturbances in the regulation of Ca^2+^ homeostasis and the presence of arrhythmogenic substrates resulting from both electrical and structural remodeling in the atria [19,20]. A study conducted using an AF model demonstrated that the GLP-1 receptor agonist can decrease the atrial effective refractory period, conduction velocity, and the propensity for AF induction [21]. Additionally, the GLP-1 receptor agonist has shown the ability to counteract the effects of heart failure on action potential duration (APD), AF inducibility, and structural remodeling, as evidenced by a reduction in the volume and fibrosis of the left atrium (LA) [22]. GLP-1 receptor agonist exhibits cardioprotective effects by reducing hyperglycemia-induced cardiomyocyte apoptosis through the improvement of sarcoplasmic/endoplasmic reticulum Ca^2+^ ATPase (SERCA) function [23]. Another study demonstrated that the GLP-1 receptor agonist significantly reduces the phosphorylation of the ryanodine receptor (RyR) at S2814. This decrease in RyR phosphorylation may suppress triggered activity and atrial arrhythmogenesis by reducing Ca^2+^ leakage, indicating the potential antiarrhythmogenic properties of the GLP-1 receptor agonist [24]. Although the direct effects of the GLP-1 receptor agonist on the pathogenesis of AF are still uncertain, it is known that the pulmonary veins (PVs) serve as primary triggers for AF [25,26], and Ca^2+^ dysregulation plays a crucial role in PV arrhythmogenesis [19,27]. Hence, this study aimed to investigate the impact of the GLP-1 receptor agonist on PV arrhythmogenesis and explore the underlying mechanisms that may explain this relationship.

## 2. Results

### 2.1. Electrophysiological Effects of the GLP-1 Receptor Agonist on PVs and Sinoatrial Node (SAN)

**Figure 1:**
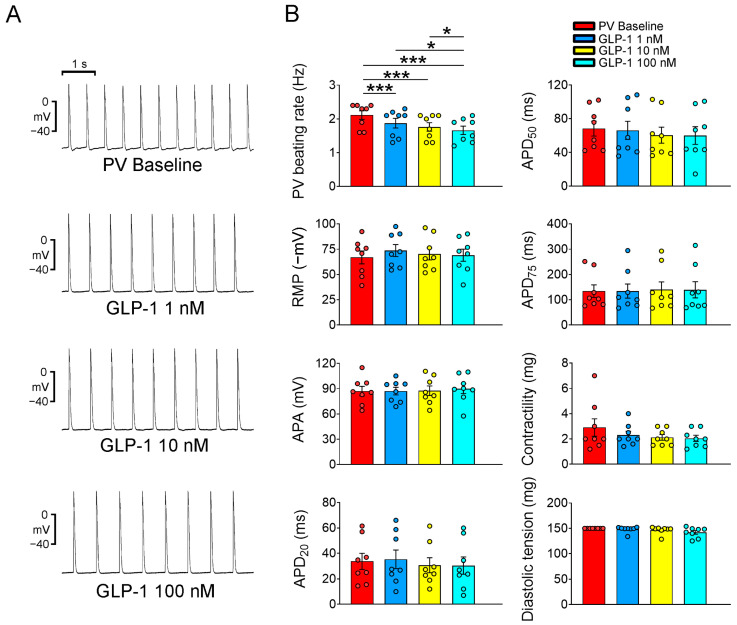
Electrophysiological effects of the glucagon-like peptide-1(GLP-1) receptor agonist on pulmonary veins (PVs). (**A**) Tracings of PVs (*n* = 8) are shown before and after treatment with GLP-1 (7-36) amide at different concentrations (1, 10, and 100 nM). (**B**) The graph displays the average beating rate, action potential parameters, contractile force, and diastolic tension of PVs before and after GLP-1 (7-36) amide treatment at the same concentrations (1, 10, and 100 nM). Statistical analysis reveals significant differences as indicated by * *p* < 0.05 and *** *p* < 0.005 compared to the baseline measurements.

**Figure 2:**
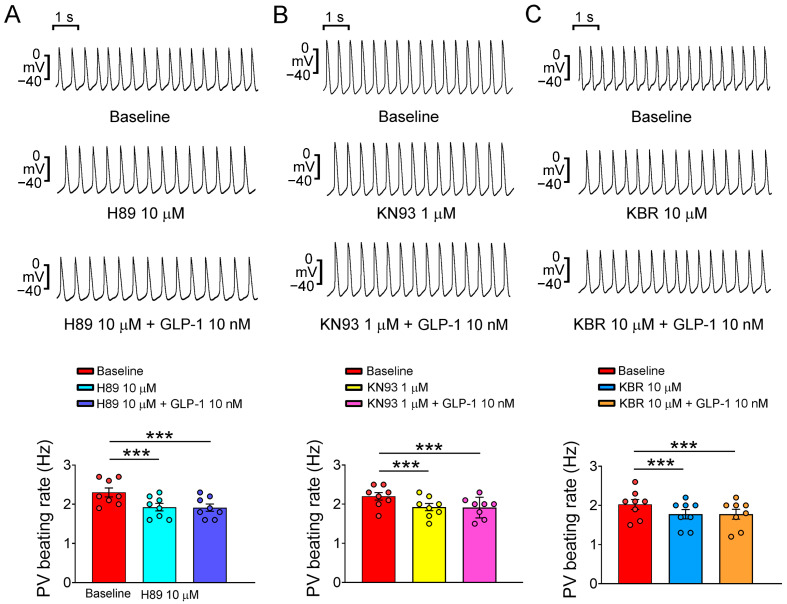
Effects of inhibition of protein kinase A, Ca^2+^/calmodulin-dependent protein kinase II, and Na^+^/Ca^2+^ exchanger on glucagon-like peptide-1 (GLP-1) receptor agonist-related electrophysiological changes in the pulmonary veins (PVs). (**A**) The upper panels show tracings of PVs (*n* = 8) at baseline, after the treatment with H89 (10 μM), and cotreatment with H89 (10 μM) and GLP-1 (7-36) amide at 10 nM. The lower panel displays the average beating rate of PVs in response to these treatments. (**B**) The upper panels present tracings of PVs (*n* = 8) at baseline, after the treatment with KN93 (1 μM), and cotreatment with KN93 (1 μM) and GLP-1 (7-36) amide at 10 nM. The lower panel shows the average beating rate of PVs following these treatments. (**C**) The upper panels illustrate tracings of PVs (*n* = 8) at baseline, after the treatment with KB-R7943 (10 μM), and cotreatment with KB-R7943 (10 μM) and GLP-1 (7-36) amide at 10 nM. The lower panel indicates the average beating rate of PVs upon these cotreatments. Statistical analysis reveals significant differences as indicated by *** *p* < 0.005 compared to the baseline measurements.

**Figure 3:**
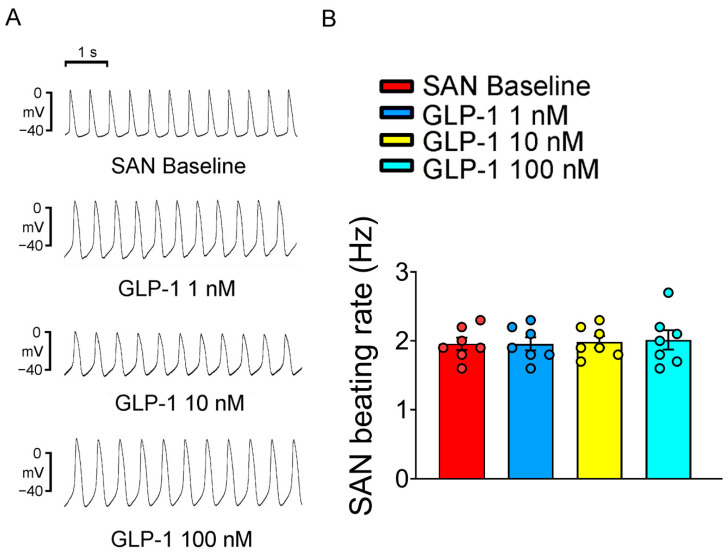
Electrophysiological effects of glucagon-like peptide-1 (GLP-1) receptor agonist on sinoatrial node (SAN). (**A**) Tracings of SAN (*n* = 7) are displayed before and after treatment with GLP-1 (7-36) amide at different concentrations (1, 10, and 100 nM). (**B**) The graph presents the average beating rate of SAN before and after GLP-1 (7-36) amide treatment at the same concentrations (1, 10, and 100 nM).

In this study, we conducted action potential recordings of PV and SAN tissue preparations. These tissues were treated sequentially with GLP-1 (7-36) amide, a GLP-1 receptor agonist, at various concentrations (1, 10, and 100 nM) for a duration of 20 min. The primary objective of this investigation was to examine the acute electrophysiological effects of GLP-1 (7-36) amide on PV and SAN tissues. As shown in Figure 1, the GLP-1 (7-36) amide exhibited a concentration-dependent effect on reducing the beating rate of the PVs. At concentrations of 1, 10, and 100 nM, the GLP-1 (7-36) amide progressively decreased the PV beating rate, suggesting a potential role of the GLP-1 (7-36) amide in modulating PV activity. In the study, the GLP-1 (7-36) amide was evaluated for its effects on various electrophysiological parameters in PV tissues. However, the administration of the GLP-1 (7-36) amide at concentrations of 1, 10, and 100 nM did not result in any significant alterations in action potential amplitude (APA), resting membrane potential (RMP), APD at repolarization levels of 20% (APD_20_), 50% (APD_50_), and 75% (APD_75_) of the APD, contractility, or diastolic tension of the PVs. Moreover, GLP-1 (7-36) amide did not induce the occurrence of early afterdepolarization (EAD), delayed afterdepolarization (DAD), or burst firing of the PVs at any of the tested concentrations (1, 10, or 100 nM). When PV tissues were pretreated with specific inhibitors, such as H89 (10 μM, an inhibitor of protein kinase A, PKA), KN93 (1 μM, an inhibitor of Ca^2+^/calmodulin-dependent protein kinase II, CaMKII), and KB-R7943 (10 μM, an inhibitor of Na^+^/Ca^2+^ exchanger, NCX), the PV beating rate was reduced to a rate similar to that in the PVs treated with GLP-1 (7-36) amide (10 nM) alone (Figure 2). This suggests that these inhibitors have an impact on the PV activity. Notably, under cotreatment with H89, KN93, and KB-R7943, the administration of the GLP-1 (7-36) amide did not further reduce PV spontaneous activity. Thus, the GLP-1 receptor agonist may reduce PV spontaneous activity via the signaling pathway regulated by PKA, CaMKII or NCX activity. However, GLP-1 (7-36) amide (1, 10, and 100 nM) had a minimal impact on SAN beating rate (as shown in Figure 3).

### 2.2. Effects of GLP-1 Receptor Agonist on PV Ionic Currents and Ca^2+^ Homeostasis

**Figure 4:**
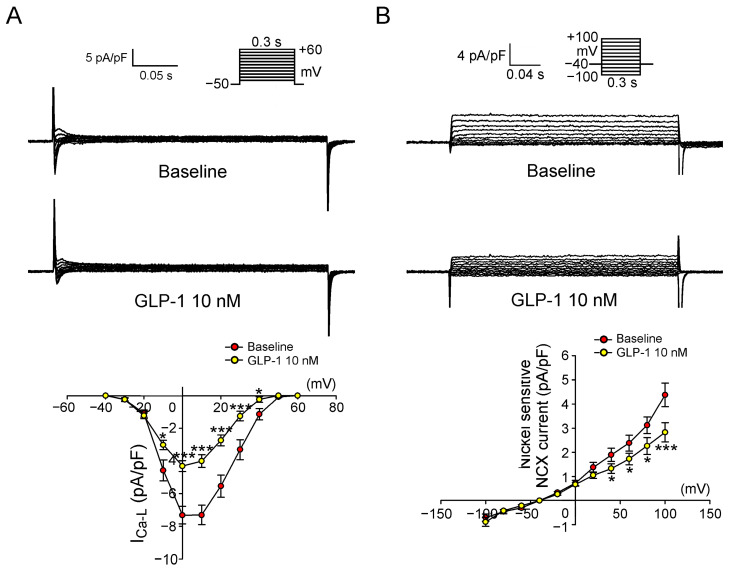
Effects of glucagon-like peptide-1 (GLP-1) receptor agonist on L-type Ca^2+^ current (I_Ca-L_) and Na^+^/Ca^2+^ exchanger current (I_NCX_) in pulmonary vein (PV) cardiomyocytes. (**A**) The upper panel displays the tracings of I_Ca-L_ before and after treatment with 10 nM GLP-1 (7-36) amide in PV cardiomyocytes (*n* = 9). The lower panel shows the current–voltage relationship of I_Ca-L_ in response to the GLP-1 (7-36) amide treatment. Insets in the current traces depict different clamp protocols used. (**B**) The upper panel illustrates the tracings of I_NCX_ before and after treatment with 10 nM GLP-1 (7-36) amide in PV cardiomyocytes (*n* = 10). The lower panel presents the current–voltage relationship of I_NCX_ following the GLP-1 (7-36) amide treatment. Insets in the current traces show various clamp protocols used. Statistical analysis indicates significant differences as denoted by * *p* < 0.05 and *** *p* < 0.005 compared to the baseline measurements.

**Figure 5:**
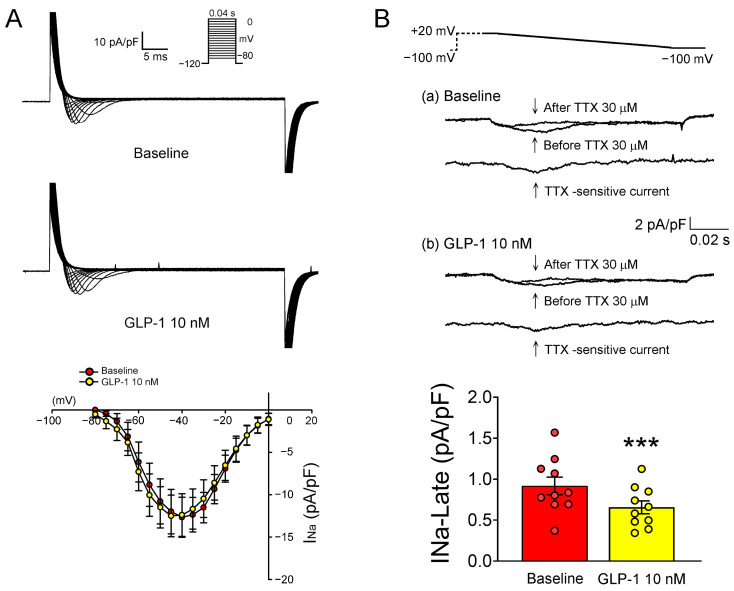
Effects of the glucagon-like peptide-1 (GLP-1) receptor agonist on Na^+^ current (I_Na_) and late Na^+^ current (I_Na-Late_) in pulmonary vein (PV) cardiomyocytes. (**A**) The upper panel presents the tracings of I_Na_ before and after treatment with 10 nM GLP-1 (7-36) amide in PV cardiomyocytes (*n* = 14). The lower panel shows the current–voltage relationship of I_Na_ following the GLP-1 (7-36) amide treatment. Insets in the current traces depict different clamp protocols used. (**B**) The upper panel illustrates the tracings of I_Na-Late_ before and after treatment with 10 nM GLP-1 (7-36) amide in PV cardiomyocytes (*n* = 10). The lower panel presents the current–voltage relationship of I_Na-Late_ following the GLP-1 (7-36) amide treatment. Insets in the current traces show various clamp protocols used. Statistical analysis indicates significant differences, as denoted by *** *p* < 0.005 compared to the baseline measurements.

Figure 4 presents the tracings and current-voltage relationships of L-type Ca^2+^ current (I_Ca-L_) and NCX current (I_NCX_) in PV cardiomyocytes before and after treatment with 10 nM GLP-1 (7-36) amide. Following GLP-1 (7-36) amide treatment, the current density of I_Ca-L_ in PV cardiomyocytes was observed to decrease compared to the pre-treatment levels. Similarly, PV cardiomyocytes displayed a reduced I_NCX_ after 10 nM GLP-1 (7-36) amide treatment compared to their state before treatment. Additionally, Figure 5 showcases the tracings and current-voltage relationships of Na^+^ current (I_Na_) and late Na^+^ current (I_Na-Late_). Notably, the administration of 10 nM GLP-1 (7-36) amide did not induce any changes in I_Na_. However, PV cardiomyocytes exhibited a decreased I_Na-Late_ after 10 nM GLP-1 (7-36) amide treatment compared to their condition before the treatment.

**Figure 6:**
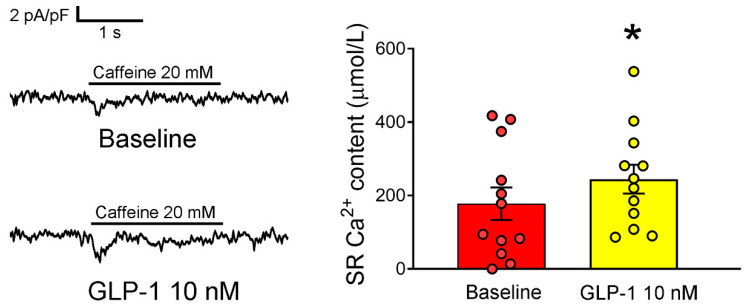
Effects of glucagon-like peptide-1 (GLP-1) receptor agonist on sarcoplasmic reticulum (SR) Ca^2+^ content in pulmonary vein (PV) cardiomyocytes. Tracings of SR Ca^2+^ content in PV cardiomyocytes (**left panel**) and corresponding average data (**right panel**) before and after 10 nM GLP-1 (7-36) amide treatment are presented (*n* = 12). Statistical analysis indicates a significant difference (* *p* < 0.05) in SR Ca^2+^ content after GLP-1 (7-36) amide treatment compared to baseline levels.

Furthermore, our observations indicated a significant increase in sarcoplasmic reticulum (SR) Ca^2+^ content in PV cardiomyocytes following treatment with GLP-1 (7-36) amide. This increase was assessed by measuring the integration of I_NCX_ after caffeine treatment and comparing it to the SR Ca^2+^ content in PV cardiomyocytes before administering 10 nM GLP-1 (7-36) amide (as depicted in Figure 6).

## 3. Discussion

This study is the first to demonstrate the acute impacts of GLP-1 receptor agonist on various aspects of PVs, including their electrical activities, ionic characteristics, and intracellular Ca^2+^ homeostasis. Furthermore, the research sheds light on the signaling mechanisms that potentially underlie these acute effects of the GLP-1 receptor agonist on PVs. Considering the crucial role of PVs as the primary triggers of AF, the findings from this study strongly indicate that the GLP-1 receptor agonist could play a critical role in regulating AF triggers.

In this study, we observed that GLP-1 receptor agonist exhibited a concentration-dependent effect on PVs by reducing their spontaneous activities. The extent of this reduction correlated with the dosage of GLP-1 receptor agonist used. Notably, the inhibitory impact of GLP-1 receptor agonist on PVs was found to be lessened when PKA, CaMKII, and NCX were inhibited, indicating that these signaling pathways play a role in mediating the effects of GLP-1 receptor agonist. Given that PVs are known as primary triggers of AF and are crucial for its maintenance [25,26,28], these results indicate that the suppression of PV automaticity by GLP-1 receptor agonist might possess substantial importance in mitigating PV arrhythmogenic activities. These arrhythmogenic activities are characterized by enhanced automaticity and the induction of microreentry, both of which are key factors in the initiation and perpetuation of AF [29,30]. Therefore, by reducing PV arrhythmogenesis, the GLP-1 receptor agonist may lead to a potential reduction in the genesis of AF.

Through our study, we have uncovered valuable insights into the effects of the GLP-1 receptor agonist on the beating rate of the SAN. Despite clinical observations indicating that the GLP-1 receptor agonist can accelerate resting heart rate in patients with diabetes [13], our specific investigation on isolated SAN revealed a contrasting result. We found that the GLP-1 receptor agonist had little direct effect on the beating rate of the SAN. Our finding indicates that the GLP-1 receptor agonist does not directly exert a positive chronotropic effect on isolated SAN, suggesting that autonomic nervous activity plays a critical role in heart rate acceleration for patients receiving GLP-1 receptor agonist treatment.

Theoretical considerations suggest that an increase in heart rate may raise the vulnerability to tachyarrhythmia [14]. In the context of AF, the activation of the sympathetic nervous system has been identified as a significant contributor to both the initiation and perpetuation of AF [31]. The PVs are richly innervated by the autonomic nervous system, and its stimulation can enhance PV arrhythmogenesis [32]. Furthermore, the administration of isoproterenol has been found to accelerate spontaneous activity within the PVs and trigger EADs and DADs [33,34]. Ca^2+^ dysregulation plays a critical role in this process. The stimulation of β1 adrenergic receptors leads to the activation of cyclic adenosine monophosphate-dependent PKA and CaMKII pathways, which in turn phosphorylate various Ca^2+^ regulatory proteins, including Ca^2+^ channels, Na^+^ channels, NCX, phospholamban (PLB), and RyR [35,36,37,38,39]. This aberrant intracellular Ca^2+^ homeostasis creates a conducive environment for PV arrhythmogenesis [40]. In our previous study [24], we found that the GLP-1 (7-36) amide had the capability to enhance Ca^2+^ transients; however, it’s important to note that these findings were observed in HL-1 cells and not in PV cardiomyocytes. Furthermore, our current research has revealed that these agonists also elevate the SR Ca^2+^ content in PV cardiomyocytes, potentially leading to an increased open probability of RyR, resulting in spontaneous diastolic Ca^2+^ release and triggering AF activity [41]. However, in this study, we found that GLP-1 receptor agonist reduced PV beating rate in a concentration-dependent manner in PV tissue preparations and reduced I_Ca-L_ and I_NCX_ in PV cardiomyocytes. Under cotreatment with H89, KN93, and KB-R7943, the administration of the GLP-1 (7-36) amide did not further reduce PV spontaneous activity. Accordingly, these findings indicate that the effects of GLP-1 receptor agonist on I_Ca-L_ and I_NCX_ could not be further suppressed under the inhibition of PKA, CaMKII, and NCX. Because I_Ca-L_ and I_NCX_ play key roles in PV electrical activity, these findings suggest that the GLP-1 receptor agonist may reduce the stress caused by excess adrenergic stimulation and even exert an antiarrhythmic effect on PVs. However, GLP-1 receptor agonists have been shown to activate PKA signaling in cultured cells [42,43]. In the present study, acute treatment of H89 mimics the effect of GLP-1 (7-36) amide, implying that the GLP-1 receptor agonist inhibits PKA activity in PV cardiomyocytes. These inconsistent findings may be related to the differences among various GLP-1 receptor agonists or caused by different experimental settings, whereas the GLP-1 (7-36) amide in this study was administered for a short period in primary isolated PV tissue preparations or single cells. Further investigations are warranted to dissect the precise molecular pathways underlying the acute effects of GLP-1 receptor agonists and how they intertwine with PKA signaling.

Several common pathological conditions in diabetes, such as heart failure, hypoxia, and an exposure to ischemic metabolites and reactive oxygen species, can cause Ca^2+^ overload through increased I_Na-Late_ [44]. This, in turn, induces spontaneous SR Ca^2+^ release and reverses NCX, which then triggers EAD and DAD [45]. An increase in I_Na-Late_ has been shown to induce sustained triggered activity in atrial cardiomyocytes [46], and the inhibition of I_Na-Late_ has been demonstrated to suppress triggered activity in PVs [47]. As depicted in Figure 5, our study demonstrates that the GLP-1 receptor agonist did not alter I_Na_ but reduced I_Na-Late_ in PV cardiomyocytes. Accordingly, our findings suggest that the GLP-1 receptor agonist may suppress PV arrhythmogenesis by reducing I_Na-Late_.

Cardiovascular risk factors and comorbidities, including diabetes and obesity, significantly increase the risk of AF development, and management of these concomitant cardiovascular risk factors and comorbidities have shown reduction of AF burden and recurrence [1,48]. In addition to glucose-dependent stimulation of insulin secretion and inhibition of glucagon secretion, the GLP-1 receptor agonist delays gastric emptying and suppresses appetite [8]. Accordingly, the GLP-1 receptor agonist not only provides glycemic control but also weight loss benefits, both of which reduce the risk of AF development and recurrence. Our study indicates that the GLP-1 receptor agonist reduces PV arrhythmogenesis, suggesting that the GLP-1 receptor agonist might protect diabetic patients from AF development and recurrence. The additional benefits of GLP-1 receptor agonist on AF may be considered, especially in diabetic AF patients who are poorly responsive, intolerant, or contraindicated to antiarrhythmic drugs.

This study has some limitations. First, we observed that the GLP-1 receptor agonist exerted acute effects on PV automaticity and Ca^2+^ homeostasis, suggesting that the GLP-1 receptor agonist may contribute to the attenuation of PV arrhythmogenesis and lead to the suppression of AF. However, the study was conducted over a short period of time, which may not capture the long-term effects of the drug. This short duration may limit the clinical relevance of the study’s findings, as the long-term effects of the drug are unknown. Simple observations of the acute effects of the GLP-1 receptor agonist on PV tissue preparations and single PV cardiomyocytes are unlikely to yield comprehensive findings that reveal the mechanisms underlying the effects of the GLP-1 receptor agonist. A whole animal must be treated with a GLP-1 receptor agonist for an extended period to uncover the detailed mechanisms involved in PV arrhythmogenesis. Second, we observed that the GLP-1 receptor agonist reduced I_Ca-L_, I_NCX_, and I_Na-Late_ in PV cardiomyocytes, suggesting that the GLP-1 receptor agonist modulates intracellular Ca^2+^ homeostasis. Because acute GLP-1 receptor agonist treatment may not alter the expressions of RyR, SERCA, and PLB, whether the GLP-1 receptor agonist may alter the phosphorylation of RyR, SERCA, and PLB to promote Ca^2+^ homeostasis is unclear. Third, in the study, we used different methods (conventional microelectrodes and whole-cell patch clamp) to measure electrophysiological parameters, which could introduce variability and may not be directly compared.

## 4. Materials and Methods

### 4.1. PV and SAN Tissue Preparations

The research study was conducted in adherence to the principles and guidelines outlined in the Declaration of Helsinki, which ensures the ethical conduct of medical research. Additionally, the study protocol was thoroughly reviewed and approved by the Institutional Review Board (IRB) of the National Defense Medical Center of Taiwan. The specific protocol code for the study was IACUC-23-045, and the IRB granted its approval on 24 February 2023. Male New Zealand white rabbits with a weight range of 2.5–3.5 kg were used in the experiment. They were first anesthetized with intramuscular injections of xylazine (5 mg/kg; Bayer AG, Leverkusen, Germany) and zoletil (10 mg/kg; Virbac, Carros, France). Afterward, the rabbits were euthanized using an overdose of inhaled isoflurane (5% in oxygen; Panion & BF Biotech, Taipei, Taiwan) for a duration of 10 min [49]. Following the sacrifice, the hearts of the rabbits were excised through a midline thoracotomy. Tissue preparations from the PV and SAN were obtained. PV tissue preparations (measuring 1 × 1.5 cm) were separated from the lungs at the end of the PV myocardial sleeves and from the LA at the LA–PV junction. Meanwhile, SAN tissue preparations (measuring 1 × 1.5 cm) were separated from the junction of the superior vena cava and the right atrium. Electropharmacological measurements were conducted within two hours after the separation procedures were completed. Each PV and SAN tissue preparation was superfused with normal Tyrode’s solution (T1 solution, see Table 1 for the composition of all solutions used in this study) at a constant rate of 3 mL/min and maintained at 37 °C. The normal Tyrode’s solution was saturated with a mixture of 3% CO_2_ and 97% O_2_, and its pH was adjusted to 7.4 using NaOH.

### 4.2. Electrophysiological Study in PV and SAN Tissue Preparations

To record the transmembrane action potentials, machine-pulled glass capillary microelectrodes filled with a solution containing 3 mol/L of KCl were used [49]. These microelectrodes were connected to an FD223 electrometer (World Precision Instruments, Sarasota, FL, USA) under a tension of 150 mg. The electrophysiological signals were displayed on a Gould 4072 oscilloscope (Gould Electronics, Eichstetten, Germany) and simultaneously recorded with a Gould TA11 recorder (Gould Electronics). Electrical stimuli were applied using a Grass S88 stimulator (Grass Instruments, Norfolk, MA, USA) and a Grass SIU5B stimulus isolation unit (Grass Instruments) at a frequency of 2 Hz to measure the action potential parameters. The following action potential parameters were measured: APA: Calculated as the difference between the peak action potential and the RMP; APD: Measured at repolarization levels of 20%, 50%, and 75% of the total APD and designated as APD_20_, APD_50_, and APD_75_, respectively. The PV and SAN tissue preparations were treated sequentially with different concentrations (1, 10, and 100 nM) of GLP-1 (7-36) amide (American Peptide Company, Sunnyvale, CA, USA) in normal Tyrode’s solution for 20 min to investigate the electrophysiological effects of GLP-1 (7-36) amide. Additionally, H89, KN93, and KB-R7943 were infused to examine the role of PKA, CaMKII, and NCX signaling pathways in the electrophysiological effects of GLP-1 (7-36) amide in the PVs. PV beating was measured at baseline, after treatment with H89 (10 μM) alone, KN93 (1 μM) alone, KB-R7943 (10 μM) alone, as well as the cotreatment of GLP-1 (7-36) amide (10 nM) with H89 (10 μM), KN93 (1 μM), or KB-R7943 (10 μM).

### 4.3. Single PV Cardiomyocyte Isolation

Single PV cardiomyocytes from rabbits were isolated using an enzymatic dissociation method, as previously described [50]. The hearts of the rabbits were excised and mounted on a Langendorff apparatus, where they were superfused with oxygenated normal Tyrode’s solution in an antegrade manner at 37 °C. Once the hearts were cleared of blood, the perfusate was replaced with oxygenated Ca^2+^-free Tyrode’s solution (T2 solution, see Table 1) containing 300 unit/mL of type I collagenase (Sigma-Aldrich, St. Louis, MO, USA) and 0.25 unit/mL of type XIV protease (Sigma-Aldrich) for a duration of between 8 and 12 min. Following the enzymatic digestion, the PVs were excised and then gently shaken in 50 mL of oxygenated Ca^2+^-free Tyrode’s solution until single PV cardiomyocytes were obtained. These isolated PV cardiomyocytes were subsequently placed and stabilized in oxygenated normal Tyrode’s solution for a minimum of 30 min before commencing the whole-cell patch clamp experiments. This stabilization period allowed the cells to acclimate to the experimental conditions and ensured reliable and consistent measurements during the whole-cell patch clamp experiments.

### 4.4. Ionic Current Measurements

Following the protocol outlined in a previous study [49], whole-cell patch clamp recordings of single PV cardiomyocytes were performed before and after the administration of GLP-1 (7-36) amide (10 nM). The experiments were conducted at a controlled temperature of 35 °C ± 1 °C. Ionic currents were measured approximately 3 to 5 min after achieving rupture or perforation of the cell membrane to ensure minimal decay in ion channel activity. To determine the total cell capacitance, a small hyperpolarizing step was applied at the beginning of each experiment. The step ranged from a holding potential of −50 mV to a test potential of −55 mV and was delivered for 80 ms. In both the current-clamp and voltage-clamp modes, ionic currents were measured to assess the electrophysiological effects of GLP-1 (7-36) amide on the single PV cardiomyocytes. The aim was to observe changes in ion channel activity and membrane potential dynamics after the application of GLP-1 (7-36) amide at the given concentration.

The I_Na_ was recorded while depolarizing the membrane potential from a holding potential of −120 mV to test potentials ranging from −80 to 0 mV in 10-mV increments. Each depolarization was applied for 40 ms at a frequency of 3 Hz, and the experiments were conducted at a temperature of 25 ± 1 °C. The external solution used for these recordings was composed of E1 solution (see Table 1), and its pH was adjusted to 7.3 using KOH. On the other hand, the micropipettes used in the patch clamp recordings were filled with a M1 solution (see Table 1), and its pH was adjusted to 7.3 using CsOH.

The I_Na-Late_ was recorded using a step/ramp protocol at room temperature. The protocol involved stepping the membrane potential from −100 mV to +20 mV for 100 ms and then ramping it back to −100 mV over 100 ms. The external solution used for these recordings was composed of E2 solution (see Table 1), and its pH was adjusted to 7.4 with NaOH. The micropipettes used in the patch clamp recordings were filled with a M2 solution (see Table 1), and its pH was adjusted to 7.3 with NaOH. To ensure adequate clamping of the cell currents, a 5- to 10-min equilibration period was allowed for dialysis. During this period, the tetrodotoxin (30 µM)-sensitive portion of the current traces was measured when the voltage was ramping back to −100 mV. This specific current component represents the I_Na-Late_.

The I_Ca-L_ was measured as an inward current activated by depolarization from −50 mV to test potentials (ranging from −40 to +60 mV). The current was applied for 300 ms at a frequency of 0.1 Hz using the perforated patch-clamp mode with amphotericin B. For these recordings, the external solution used was composed of E3 solution (see Table 1), with its pH adjusted to 7.4 using NaOH. Then, 4-aminopyridine (2 mM) and Tetrodotoxin (10 μM) were added to the external solution to block I_Na_ and transient outward K^+^ current, respectively. The micropipettes used in the patch clamp recordings were filled with a M3 solution (see Table 1), and its pH was adjusted to 7.2 with CsOH.

The I_NCX_ was measured by applying depolarizing pulses ranging from −100 to +100 mV from a holding potential of −40 mV. Each pulse lasted for 300 ms and was delivered at a frequency of 0.1 Hz. The amplitudes of I_NCX_ were quantified as the 10-mM nickel-sensitive current. For these recordings, the external solution used was composed of E4 solution (see Table 1), and its pH adjusted to 7.4 using NaOH. The micropipettes used in the patch clamp recordings were filled with a M4 solution (see Table 1), and its pH was adjusted to 7.25 with CsOH.

### 4.5. Measurements of SR Ca^2+^ Content

After achieving a steady state of Ca^2+^ transients through repetitive pulses from −40 to 0 mV (at a frequency of 1 Hz for 5 s), the SR Ca^2+^ content was assessed. This measurement was performed by integrating the I_NCX_ following the application of caffeine (at a concentration of 20 mM) within a 0.5-s window during rest. To induce SR Ca^2+^ release, the membrane potential was clamped to −40 mV [51]. The calculation for determining the total SR Ca^2+^ content, expressed in millimoles (mM) of cytosol, was based on the following equation: SR Ca^2+^ content = [(1 + 0.12) (Ccaff/F × 1000)]/(Cm × 8.31 × 6.44). In this equation: Ccaff stands for the caffeine concentration, F represents Faraday’s number, Cm denotes the membrane capacitance, the cell surface to volume ratio is given as 6.44 pF/pL. By utilizing this formula, we were able to quantitatively determine the SR Ca^2+^ content in the cytosol during the experimental conditions described [52,53].

### 4.6. Statistical Analysis

Continuous variables were presented as mean ± standard error to provide a descriptive overview of the data. To assess the differences before and after GLP-1 receptor agonist treatment, and the treatment with H89, KN93, or KB-R7943, as well as the cotreatment of GLP-1 (7-36) amide (10 nM) with H89 (10 μM), KN93 (1 μM), or KB-R7943 (10 μM) on the results, either a paired t-test or a one-way repeated measures analysis of variance (ANOVA) was employed. Following ANOVA, the Bonferroni post hoc test was used to further analyze specific differences between groups. Statistical significance was indicated by a *p*-value less than 0.05, denoting that the observed results were unlikely to have occurred by chance and were considered statistically meaningful.

## 5. Conclusions

GLP-1 receptor agonist reduced PV automaticity, which was attenuated by H89, KN93, and KB-R7943; these findings suggest that the interaction between GLP-1 receptor agonist and PV automaticity is associated with the PKA, CaMKII, and NCX signaling pathways. GLP-1 receptor agonist reduced I_Ca-L_, I_NCX_, and I_Na-Late_ in PV cardiomyocytes, which indicates GLP-1 receptor agonist modulates intracellular Ca^2+^ homeostasis.

## Figures and Tables

**Table 1:** Composition of external and pipette solutions.

	T1	T2	E1	E2	E3	E4	M1	M2	M3	M4
NaCl (mM)	137	137	5	130		140	5			20
NaHCO_3_ (mM)	15	15								
NaH_2_PO_4_ (mM)	0.5	0.5								
KCl (mM)	4	4								
CaCl_2_ (mM)	2.7		1.8	1	1.8	2				1.75
MgCl_2_ (mM)	0.5	0.5	2	1	0.5	1		1	1	0.4
Dextrose (mM)	11	11								
CsCl (mM)			133	5	133		133	130	130	110
Nifedipine (mM)			0.002							
HEPES (mM)			5	10	10	5	5	5	10	10
MgATP (mM)							5		5	5
EGTA (mM)							10	10	10	
TEACl (mM)					20		20			20
Glucose (mM)			5	10	10	10				5
Na_2_ATP (mM)								4		
NaGTP (mM)									0.1	
Na_2_phosphocreatine (mM)									5	
Strophanthidin (mM)						10				
Nitrendipine (mM)						10				
niflumic acid (mM)						100				
BAPTA (mM)										5
Tetrodotoxin (mM)					10					
4-aminopyridine (mM)					2					

HEPES = 4-(2-hydroxyethyl)-1-piperazineethanesulfonic acid; EGTA = ethylene glycol-bis (β-aminoethyl ether)-*N*,*N*,*N*′,*N*′-tetraacetic acid; TEACl = tetraethylammonium chloride; BAPTA = 1,2-bis (o-aminophenoxy) ethane-*N*,*N*,*N*′,*N*′-tetraacetic acid.

## Data Availability

Supporting data or materials that validate the findings of this study can be accessed through the corresponding author upon reasonable request.
